# Transforming Food Systems for the Prevention of Childhood Overweight and Obesity in the Gulf Region †

**DOI:** 10.3390/nu18203435

**Published:** 2026-10-20

**Authors:** Nada Alnaji, Ghadeer S. Aljuraiban, Kate Heneghan, Najla Alorayyidh, Aashima Garg, Jumana Haj-Ahmad, Sarah Zahr, Qureshi Abdul Baseer, Rasha Alfawaz

**Affiliations:** 1 Public Health Programs and Policies, Gulf Center for Disease Prevention and Control, Riyadh 12512, Saudi Arabia; n.alnaji@gulfcdc.org (N.A.); r.alfawaz@gulfcdc.org (R.A.); 2 Department of Community Health Sciences, College of Applied Medical Sciences, King Saud University, Riyadh 12371, Saudi Arabia; galjuraiban@ksu.edu.sa; 3 Center of Excellence, United Nations Children’s Fund (UNICEF), Amman 11821, Jordan; kheneghan@unicef.org (K.H.); agarg@unicef.org (A.G.); sarahzahr00@gmail.com (S.Z.); 4 Gulf Area Office, United Nations Children’s Fund (UNICEF), Riyadh 94623, Saudi Arabia; jhajahmad@unicef.org; 5 Eastern Mediterranean Regional Office, World Health Organization (WHO), Khartoum 2234, Sudan; qureshiab@who.int

**Keywords:** childhood overweight and obesity, gulf cooperation council, food environment, food systems, policy, marketing restrictions, infant and young child feeding, school-age children and adolescents, governance and accountability

## Abstract

**Background/Objectives**: The prevalence of childhood overweight and obesity is increasing rapidly across the Gulf Cooperation Council (GCC), threatening public health and economic sustainability. Drivers include increased availability and consumption of ultra-processed foods and sugar-sweetened beverages, physical inactivity, and pervasive marketing of unhealthy foods. This study used a secondary, framework-guided narrative synthesis of two regional reports to map child-relevant policy coverage, examine reported implementation barriers, and identify priorities for action across the six GCC countries. **Methods**: We used two comprehensive regional reports by the Gulf Center for Disease Prevention and Control (Gulf CDC), United Nations Children’s Fund (UNICEF), and World Health Organization. The analysis synthesizes 103 policy-related documents and 32 key-informant interviews across six Gulf countries. **Results**: Our findings reveal a policy environment with significant gaps despite growing political commitment and a wide range of national strategies: three countries have adopted front-of-pack nutrition labeling, on a voluntary basis; marketing restrictions remain partially implemented, particularly on digital platforms. Stakeholders highlighted fragmented governance and limited accountability. This directly affects service delivery. While all countries have school-based interventions and infant and young child feeding strategies, their impact is weakened by inconsistent funding and implementation. **Conclusions**: Using UNICEF’s Transforming Children’s Food Systems approach, we propose a GCC-wide roadmap in three action areas: (1) improving children’s foods; (2) improving children’s food environments; and (3) improving children’s food practices. This roadmap offers a strategic path for the GCC to accelerate action towards protecting the health and well-being of all children in the Gulf and offering valuable lessons for global policy and practice.

## 1. Introduction

Childhood overweight and obesity are a growing global threat. Children affected by overweight are more likely to become overweight as adults and will be at greater risk of developing non-communicable diseases (NCDs), such as diabetes, heart disease, and cancer later in life [1]. Overweight and obesity also have significant indirect economic costs [2], predicted to reach $4.3 trillion worldwide by 2035, accounting for about 3% of the entire global Gross Domestic Product [3].

The Gulf Cooperation Council (GCC) countries, comprising the United Arab Emirates (UAE), Kingdom of Bahrain, Kingdom of Saudi Arabia, Sultanate of Oman, State of Qatar, and State of Kuwait, account for some of the highest rates of overweight and obesity globally [4]. According to estimates in 2024, between 8.4–10.1% of children under 5 in the GCC are overweight, exceeding the global average of 5.5% [5]. The prevalence is even more alarming among children and adolescents aged 5–19 years where the range is between 39–47%, which is well above the global average of 20% [6,7]. Among adult women, the prevalence of overweight ranges between 69–82% [8].

This public health challenge is driven by a complex web of factors unique to the region’s rapid development, primarily the presence of an obesogenic environment. This environment promotes high consumption of energy-dense, ultra-processed foods (UPF) and sugar-sweetened beverages (SSBs), and is compounded by pervasive food marketing, rapid urbanization, and car-dependent infrastructure [9,10,11,12]. UPFs are industrial formulations containing ingredients or additives not commonly used in domestic food preparation. Examples include soft drinks, many packaged sweet and savory snacks, and some ready-to-eat meals. These products are often high in sugar, salt or fat [13]. Early-life determinants, including high rates of maternal obesity, low birth weight, and suboptimal breastfeeding practices, further contribute to the increased risk of overweight later in life [14,15,16]. This burden poses challenges for achieving the goals of the GCC’s national Vision frameworks and their commitment to Sustainable Development Goals (SDG 2.2; SDG 3.4) [17].

Policies grounded in food systems transformation, which shape the broader food environment through laws, regulations, and strategies, have the greatest potential to halt this rise. The United Nations Children’s Fund (UNICEF) Middle East and North Africa (MENA) Nutrition Strategic Direction 2030 outlines a multi-systems approach to address all forms of malnutrition among children, adolescents and women, and identifies prevention of childhood overweight as a priority for accelerated action in GCC countries [8]. Similarly, the World Health Organization’s (WHO) Regional Office for the Eastern Mediterranean Nutrition Strategy 2020–2030 sets the policy frame, with a 2023 guideline urging protections from harmful food marketing to children and complementary guidance on front-of-pack labeling and fiscal policies [18,19].

Furthermore, previous regional research has examined obesity-prevention policies, intersectoral coordination and health-promotion capacity, identifying challenges in implementation and evaluation [20]. A systematic review of school-based weight-related interventions in GCC countries also identified methodological limitations that restricted conclusions about their effectiveness [21]. These contributions provide evidence on broad policy challenges and interventions within specific settings. An important task is to connect documented policies across domains relevant to childhood obesity with evidence on the institutional and service-delivery conditions affecting their implementation. The Gulf Center for Disease Prevention and Control (Gulf CDC) [22] and UNICEF [23] regional reports provide complementary information for this purpose.

This study therefore aimed to integrate child-relevant findings from these two regional reports across the six GCC countries. Specifically, it sought to map documented policy coverage, examine reported implementation and governance barriers, and identify priorities for national action and regional collaboration. The synthesis applies UNICEF’s Food Systems for Children framework to organize findings around children’s foods, food environments and food practices [24]. Its contribution lies in bringing the broader NCD policy mapping together with the child-focused food-systems assessment, linking documented measures and stakeholder perspectives to practical recommendations. The resulting roadmap identifies priorities for strengthening childhood obesity prevention while distinguishing policy commitments from evidence of delivery and effectiveness. The recommendations also draw on a regional consultation convened by UNICEF, WHO and the Gulf CDC in Riyadh on 4–5 February 2025 [23]. The meeting brought together representatives from government, public health authorities, academia, civil society and international organizations to review existing policies and programs, identify gaps, agree on priority actions and seek commitment to their implementation. Country-group discussions informed priorities for national action and regional collaboration.

## 2. Materials and Methods

### 2.1. Analytical Approach

We conducted a secondary, framework-guided narrative synthesis of two regional reports to map child-relevant policy coverage and identify priorities for action across the six GCC countries. Specifically, we integrated findings from two comprehensive regional reports: (1) the Gulf CDC Mapping of Policies Related to Non-Communicable Diseases in the GCC Region [22] and (2) the UNICEF, Gulf CDC and WHO Report on Transforming Food Systems for the Prevention of Childhood Overweight in Gulf Countries [23].

**Figure 1:**
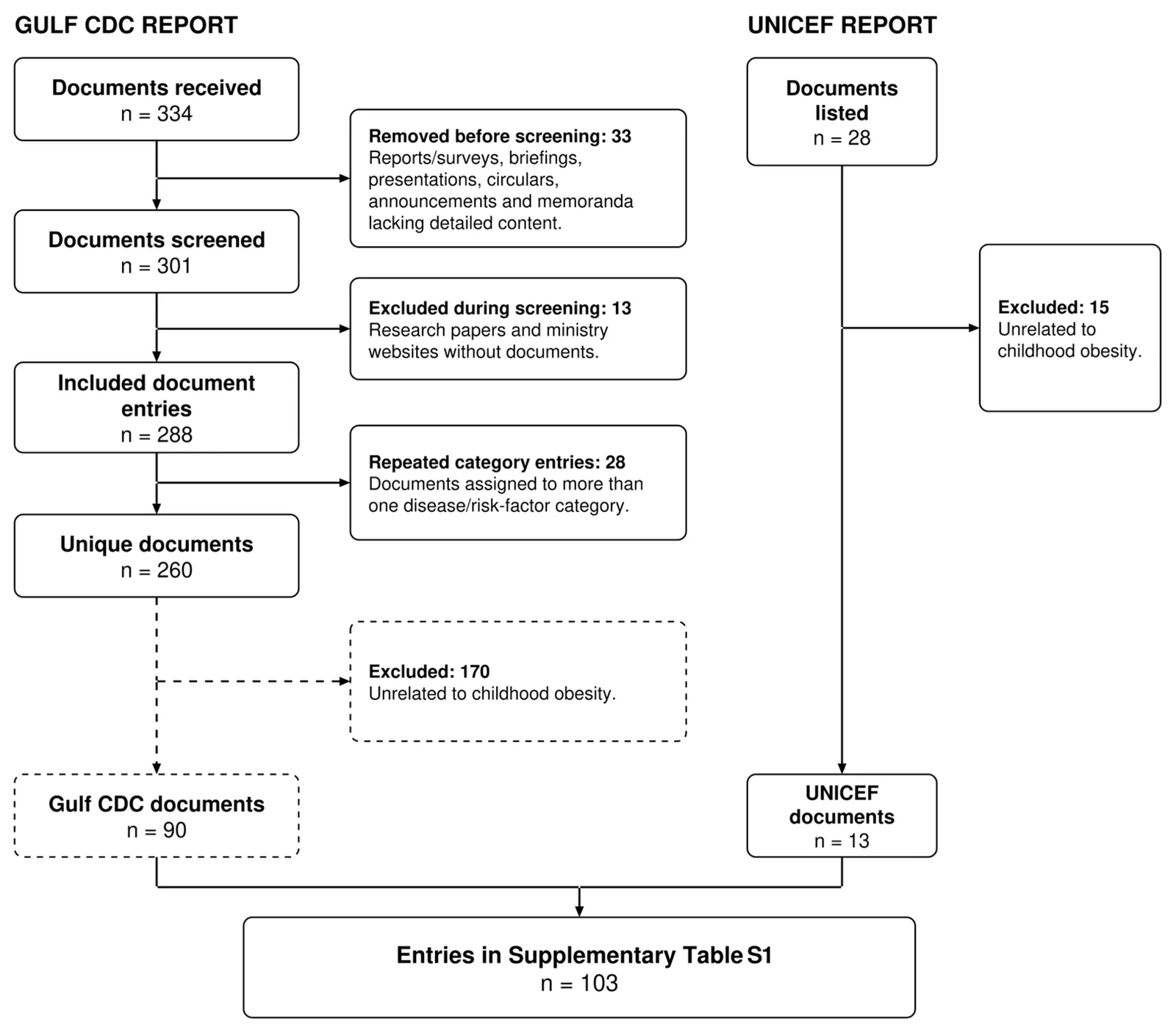
Identification and selection of policy-related entries from the Gulf CDC and UNICEF reports.

The policy inventory comprised 103 policy-related entries, including national strategies, regulatory instruments, guidelines and programs. Eligibility was based on relevance to childhood or adolescent obesity prevention and related determinants, including nutrition, infant and young child feeding, school food environments, food marketing, physical activity, urban planning and social determinants. Supplementary Table S1 identifies the included entries, and Figure 1 summarizes their identification and selection from the two source reports. The inventory and the manuscript reference list serve different purposes: the former lists the included policy-related entries, whereas the latter cites the regional reports and supporting literature. The shorter reference list therefore does not indicate that entries were excluded from the inventory.

In the original Gulf CDC mapping study, Checklist 1 collected information on document characteristics, target populations, publication dates, responsible institutions and risk factors addressed. Checklist 2 sought more detailed information on implementation, targets, effectiveness and supporting evidence. Missing information was checked against available documents and otherwise retained as missing [22]. These source-study assessments provided information for the present secondary synthesis.

The qualitative component drew on findings from 17 stakeholder interviews reported in the Gulf CDC mapping report [22] and 15 key-informant interviews described for the UNICEF report [23]. Gulf CDC interviewees represented all six GCC countries and included directors, department heads, clinicians, and technical representatives working in public health, NCD prevention, primary health care, nutrition, food regulation, youth affairs, commerce, agriculture, and local administration. For the Gulf CDC study, Permanent Communication Network members, supported by ministry experts, conducted national stakeholder mapping, and participants were selected using purposive and snowball sampling. The Gulf CDC interviews followed a structured interview guide covering institutional roles, government engagement, political commitments, sector-specific interventions, and the role of regional entities. A single interviewer conducted the interviews to maintain consistency, following training informed by a pilot test of the interview protocol. Two Gulf CDC team members also attended each interview and asked follow-up questions when necessary. Most interviews were conducted in Arabic, with two conducted in English, according to participants’ preferences. Interviews were recorded and transcribed, with translation into English for analysis.

The UNICEF study used semi-structured key-informant interviews with representatives of government ministries, public health authorities, academia, civil society organizations, and international partners. The interviews aimed to validate desk-review findings and identify contextual barriers and enablers to policy implementation and coordination. The flexible format allowed exploration of country-specific circumstances and stakeholder experiences. The report used thematic analysis to identify shared themes, challenges, and opportunities [23].

Both source studies analyzed the interview material qualitatively. The Gulf CDC study used NVivo version 15 and combined deductive coding, informed by policy-analysis frameworks, with inductive coding to identify emerging themes. Researchers double-coded a sample of transcripts and discussed discrepancies. The UNICEF study used thematic analysis to identify shared themes, challenges and opportunities.

For the present manuscript, two researchers independently reviewed information extracted from the reports and used a consensus-based matrix to categorize findings by life stage, sector and document type. UNICEF’s Food Systems for Children framework [24] provided the overarching structure, organizing policies and interventions into three action areas: improving children’s foods, improving children’s food environments and improving children’s food practices (Table 1). We narratively synthesized the reported policy-document and interview findings to describe policy coverage and implementation challenges across countries, without undertaking additional transcript coding. The lead analysts of both source reports cross-checked the synthesized findings.

Stakeholder accounts informed interpretation of institutional responsibilities and implementation barriers. The source reports did not provide a harmonized respondent-level dataset suitable for calculating pooled response frequencies or statistical associations with childhood obesity outcomes. Stakeholder perspectives were therefore treated as contextual qualitative evidence rather than estimates of the prevalence of particular views.

Descriptive country-level counts summarized documented measures across school nutrition, food marketing, front-of-pack labelling, infant and young child feeding, and physical activity (Supplementary Table S2). Each country was counted once per specified measure, using six countries as the denominator. Legal status was distinguished from implementation, monitoring and evaluation. Mandatory and voluntary components were not treated as mutually exclusive within a broader policy domain. Where the reports differed or provided insufficient information, uncertainty was retained rather than interpreted as policy absence.

For interpretation within the present synthesis, policy existence meant that a source report identified an instrument or measure. Adoption required explicit reporting of formal approval or issuance by the responsible authority, whereas implementation referred to reported delivery of the specified actions. Mandatory and voluntary described an obligation or an optional measure explicitly characterized as such in a source. Enforcement referred to reported activities to secure compliance, such as inspections, corrective actions or sanctions. Monitoring referred to ongoing collection and review of policy-specific indicators, whereas evaluation referred to a systematic assessment of policy implementation or results. Provisions for future enforcement, monitoring or evaluation were distinguished from reports that these activities had occurred. An evaluation did not, by itself, establish policy effectiveness.

The full and partial implementation labels in Table 2 were retained from the UNICEF report’s classifications [23]. That report did not specify standardized thresholds for coverage, compliance or completion of policy components. These labels therefore represent source-reported assessments rather than independently verified implementation scores and do not independently establish the extent of delivery. The Gulf CDC report provided additional information on implementation and supporting evidence. These distinctions guide interpretation of the findings in Section 3.1.

### 2.2. Ethical Approval and Informed Consent

This manuscript presents a secondary synthesis of policy documents and anonymized key-informant interview data. Institutional ethical approval for the Gulf CDC data collection, including the administration of the checklists and conduct of the key-informant interviews, was obtained from the Institutional Review Board of King Saud University, Riyadh, Saudi Arabia (approval reference: KSU-HE-24-472). The UNICEF key-informant interviews were conducted as part of a regional landscape analysis and did not undergo formal institutional review board review; verbal informed consent was obtained from interview participants. The present manuscript is a secondary synthesis of policy documents and de-identified findings and involved no new participant data collection. All interview participants provided verbal informed consent before participating.

## 3. Results

### 3.1. Policy-Implementation Gap

The country-level summary identified 103 relevant policy and strategy documents covering multiple, overlapping domains (Supplementary Table S1). Domain-specific country counts and proportions across school nutrition, food marketing, front-of-pack labelling, infant and young child feeding, and physical activity are summarized in Supplementary Table S2. The reports documented at least one school nutrition measure in each of the six countries (6/6; 100%). Measures included school food standards, restrictions on selected unhealthy items and nutrition education, with the combination varying by country.

The food environment is a primary example to illustrate this gap. Three countries (the UAE, Saudi Arabia, and Oman) have introduced front-of-pack food labelling policies. Still, they remain voluntary and are not uniformly adopted across food manufacturers, even within those countries, leading to partial integration and limited consumer impact. Similarly, restrictions on the marketing of unhealthy foods and beverages targeted at children are partial. All six countries have Infant and Young Child Feeding (IYCF) policies with varying funding allocation, implementation reach, and clarity of objectives. An additional gap concerns child-focused settings beyond schools and health facilities. Policies that align the entertainment and tourism sectors with child nutrition guidelines remain largely absent, even though food offerings in many leisure and tourism venues frequented by children are often dominated by chain fast-food vendors without clear nutritional standards or regulatory oversight. This represents an important opportunity to expand the GCC-wide Healthy Settings Initiative to include these environments and create healthier food settings for children across the region.

These regulatory weaknesses sit alongside high-risk exposures. Daily smartphone use is widespread, and 75% of parents reported increased child purchase requests after exposure to food advertisements. Frequent fast-food intake among adolescents is common, with several countries reporting approximately 24–30% of adolescents aged 13–17 consuming fast food on at least three days per week, and about half of all children in several GCC countries consume SSBs or sweets on a daily basis [22,23,24]. Table 2 summarizes reported policy coverage across the six GCC countries using the UNICEF report’s classifications. These classifications should be interpreted according to the definitions and limitations described in Section 2.1.

### 3.2. Institutions and Governance

The 32 key informant interviews identified stakeholder-perceived institutional factors contributing to policy limitations. Although a strong political commitment to address childhood overweight in all Gulf countries is present, there is limited integration across health, education, urban planning, and food sectors. For instance, cross-sectoral task forces on school nutrition remain limited or missing in several countries. Stakeholders described siloed mandates, weak accountability, unclear monitoring arrangements and variable financing, producing uneven execution even where strategies exist.

“Each sector is working in silos. We have nutrition guidelines and school health programs, but they are not aligned with broader urban planning or food supply policies.”—Public Health Authority representative, Saudi Arabia.

Furthermore, private sector and civil society involvement is often limited and not systematic. This is compounded by significant gaps in monitoring and evaluation. As the analysis shows, national surveillance of childhood overweight and obesity is inconsistent and not standardized, many programs lack built-in outcome evaluation, and regional data sharing is underdeveloped. “We often start promising programs but do not have the data systems to track their long-term impact on children’s health.”—Academic expert, Qatar.

Stakeholders also described how widely available unhealthy food marketing remains on digital platforms, which undermines national public health messaging. At the time this manuscript was drafted, the earlier Gulf Standardization Organization (GSO) framework on marketing food directed to children remained a Gulf Standard and therefore functioned as a voluntary framework. As of April 2026, however, GSO 2725:2025, Marketing Requirements of Food Directed to Children, was the current Gulf Technical Regulation, having been approved on 29 October 2025, and is mandatory at the GCC level [25]. In the UAE, Abu Dhabi introduced a mandatory policy in October 2025 prohibiting advertisements for unhealthy foods and beverages classified as SEHHI grades C, D or E across outdoor advertising media regulated by the Department of Municipalities and Transport, including billboards and digital displays. This restriction applies at the emirate level [26].

### 3.3. Service Delivery

These institutional gaps directly impact the delivery of services on the ground. For IYCF services, all countries have introduced the Baby-Friendly Hospital initiative. This initiative supports breastfeeding in facilities providing maternity and newborn services, but gaps remain in ensuring lactation support, establishing community-based counselling programs, and integrating breastfeeding promotion into routine pediatric care [27,28,29,30,31,32]. Additionally, inconsistent maternity leave policies and workplace support mean that exclusive breastfeeding rates remain low.

In schools, the reports documented measures in all six countries of some form of nutrition intervention, including nutrition standards for foods served in schools, partial bans on the sale of unhealthy items (e.g., sugary drinks, UPF snacks) via vending machines, and the integration of nutrition topics into school curricula (Supplementary Table S1). However, these strategies are voluntary in several countries and monitoring of their implementation is uneven or unclear (Table 2). In contrast, Kuwait and Qatar appear to have more structured frameworks, including oversight mechanisms for school food environments. While nutrition may be in the school curricula, physical education is not always mandatory, with timetables typically allowing only one or two short sessions per week, which is insufficient to meet WHO recommendations of at least 60 min daily of moderate-to-vigorous physical activity across the week for ages 5–17 [33]. In practice, 25% or fewer of school-age children in the UAE and Qatar meet the guideline [34,35]. Sedentary behavior is also high, with preschool children in Saudi Arabia averaging about 3.1 h/day of screen time [36] and in Qatar, more than half of children aged 5–17 years spend ≥2 h/day [35].

Most countries also incorporate physical activity goals into their national health strategies (e.g., Saudi Arabia’s “Sports for All” and Bahrain’s Strategy for Physical Activity and Healthy Diet) (Supplementary Table S1). In Saudi Arabia, the “Sports for All” federation promotes community engagement in physical activity through nationwide campaigns, public fitness events, and investments in accessible sports infrastructure and walking trails [37].

Despite initiatives to urbanize cities and create innovative approaches for weather-resistant spaces, stakeholders noted that accessibility is often limited in different geographic and socioeconomic groups. Efforts to create inclusive youth spaces (e.g., recreation centers) are underway in the UAE and Oman, but implementation varies across regions and municipalities, mainly depending on local resources, priorities, and coordination between sectors.

## 4. Discussion

### 4.1. Main Findings

The synthesis identifies strengths in the GCC’s political commitment and broad foundation of policies addressing childhood obesity. These include national nutrition and obesity-prevention strategies, and fiscal and regulatory measures such as sugar-sweetened beverage taxation and school nutrition standards. Existing school nutrition and infant and young child feeding initiatives provide platforms for preventive action. However, several gaps in current prevention efforts remain. Reported implementation gaps indicate a disconnect between policy commitments and their delivery (Table 2). The domains most relevant to prevention include food quality and affordability, food marketing, school nutrition and physical activity, and early feeding practices. Early feeding priorities include continuity of breastfeeding and complementary-feeding counselling across maternity, primary care, and community services. Prevention should also connect with family-based care for children already living with obesity. This discussion will focus on three overarching themes that explain this gap: fragmented governance, the limits of voluntary regulation, and the need for better data and monitoring.

A primary challenge is fragmented governance. As stakeholders reported, government ministries often work in silos, leading to a lack of policy coherence. This limits coordination of nutrition and health goals across sectors, including education and urban planning. Furthermore, there is a need to examine policies on nutrition and physical activity in daycare and childcare settings, because regulatory oversight can be outside the education sector and may be split across authorities. For example, in Saudi Arabia, private childcare centers are licensed and supervised by the Ministry of Human Resources and Social Development [38], and UNESCO’s Kuwait profile identifies the Ministry of Social Affairs and Labor as responsible for non-state nurseries [39]. A Saudi study illustrates the implementation of a whole-school approach. Conducted in 2022 across 20 public elementary schools in Makkah, this cross-sectional study assessed community involvement, family engagement, the physical environment and counselling services. Health-promoting schools had higher reported implementation scores than other schools, although gaps remained in community involvement, parental participation and structured mental health programs. The findings identify priorities for strengthening school health programs but do not establish effects on dietary or physical activity behaviors [40].

Addressing the identified governance gaps therefore requires shared nutrition and physical activity standards, assigned institutional responsibilities, and funded joint implementation plans. Without formal mechanisms for cross-sectoral collaboration, public health interventions remain isolated, and their potential impact is diminished.

The analysis shows that while front-of-pack labelling remains voluntary in some settings, the updated GCC framework on marketing of food directed to children has now been adopted as a mandatory Gulf Technical Regulation [25]. The remaining challenge is therefore less the absence of a regional rule and more the need for effective national implementation, enforcement, and monitoring, particularly in digital environments. Fiscal measures provide an evaluated example relevant to food affordability and purchasing. An evaluation of Saudi Arabia’s 50% carbonated drink excise tax found a 55% price increase and a 35% relative reduction in per-capita sales compared with comparable Gulf countries [41]. The price increase is consistent with the intended mechanism of reducing affordability, although the study did not establish changes in children’s intake or obesity. These findings support combining fiscal measures with enforceable marketing restrictions and healthier food provision. Clear nutrient criteria, designated enforcement responsibilities, and routine compliance checks would connect policy commitments to measurable changes in children’s food environments.

Finally, the source reports describe gaps in nutrition surveillance and policy monitoring that limit assessment of implementation, outcomes and accountability. Regional monitoring should distinguish policy adoption, implementation, and health outcomes, using indicators such as school food compliance, exposure to unhealthy food marketing, feeding-support coverage, dietary intake, physical activity, and measured weight status. Evaluation should also examine how prevention connects with obesity management. Recent evidence from Saudi Arabia illustrated the importance of moving beyond policy adoption to monitoring implementation. An evaluation of 80 primary schools in Riyadh found that, despite mandatory school-canteen guidelines, 70% of schools had average compliance, 30% had poor compliance, and none had good compliance with school-canteen guidelines; healthy-food availability also remained limited [42].

These findings reinforce the need for routine compliance monitoring and clearly defined implementation indicators alongside policy adoption. Monitoring should assess compliance with school food standards and the availability and affordability of healthy options. Evaluation should also examine whether implementation and access differ across geographic and socioeconomic settings. These indicators could guide corrective action and resource allocation.

### 4.2. GCC Roadmap Through a Children’s Food Systems Transformation Approach

The GCC can accelerate progress on child nutrition through three coordinated action areas. Action Area 1, improving children’s foods, should adopt regional food-based dietary guidelines that address the nutrition rights and needs of children, adolescents and women. Action Area 2, improving children’s food environments, should support implementation and monitoring frameworks for school food and school food environments, including the global standards for health-promoting schools [43] and restricting the marketing of unhealthy foods and beverages to children in line with the updated GSO technical regulation on marketing requirements of food directed to children. Action Area 3, improving children’s food practices, can scale up to mandate health insurance companies to cover breastfeeding counseling, consultations, and support the Baby-Friendly Hospital Initiative in facilities and communities, consistent with the WHO Comprehensive Implementation guidance [44]. Priority regional recommendations are summarized in Table 3.

### 4.3. Strengths and Limitations

A strength of this study is its coverage of all six GCC countries, providing a regional perspective on childhood obesity prevention. The synthesis integrates policy information, stakeholder perspectives, and epidemiological evidence from two complementary regional reports, allowing documented policy commitments to be considered alongside reported implementation challenges. Applying UNICEF’s Transforming Children’s Food Systems framework provides a consistent structure for examining children’s foods, food environments, and food practices. This approach supports comparison across countries and helps identify shared policy gaps, country-specific priorities, and opportunities for regional collaboration.

This study has several limitations. As a secondary synthesis, it depended on the coverage, completeness, and quality of two regional reports with different scopes and existing qualitative analyses. Inconsistent policy documentation may have resulted in the omission of relevant measures, while differences in terminology and reporting limited cross-country comparability. The available sources did not provide sufficiently comparable policy-specific evidence to calculate reliable proportions for monitoring and completed outcome evaluation across countries and domains. Incomplete reporting should not be interpreted as evidence that monitoring or evaluation was absent. Gaps in age-specific overweight and obesity estimates and breastfeeding data restricted assessment of early-life nutrition and inequalities. Stakeholder accounts provided contextual insights but reflected the perspectives of those interviewed and may have been affected by reporting bias. Translation of Arabic interview material into English may also have affected the interpretation of nuanced responses. Furthermore, the existence of a policy does not establish its implementation or effectiveness. This synthesis did not independently measure enforcement, program reach, or changes in childhood obesity attributable to individual policies. The findings therefore describe documented policy coverage and reported implementation challenges and do not establish causal effects. Policy developments beyond the period covered may also not be captured.

## 5. Conclusions

This synthesis identifies gaps between the Gulf region’s nutrition policy commitments and their reported implementation, enforcement, and monitoring. Future action should prioritize funded multisectoral implementation plans with clear institutional responsibilities and measurable targets. Specific priorities include establishing nutritional standards for foods intended for children, enforcing school food standards and procurement rules, implementing and monitoring restrictions on unhealthy food marketing, including digital marketing, and expanding breastfeeding and complementary feeding support through health and community services. These priorities address UNICEF’s three action areas: children’s foods, food environments, and food practices.

At the regional level, the Gulf CDC, working with national authorities, UNICEF, and WHO, could coordinate common indicators and periodic reporting on implementation and outcomes. Monitoring should assess school food compliance, children’s exposure to unhealthy food marketing, access to feeding support, dietary quality, and childhood overweight and obesity. Future research should evaluate whether policies reach their intended populations and improve dietary and health outcomes, including differences across age groups, socioeconomic groups, and geographic areas. This would provide evidence to guide policy adaptation and resource allocation while distinguishing policy adoption from implementation and measurable benefit.

## Figures and Tables

**Table 1:** UNICEF Food Systems for Children Action Areas.

Action Area 1: Improving Children’s Foods	Action Area 2: Improving Children’s Food Environments	Action Area 3: Improving Children’s Food Practices
1.1 Food guidelines	2.1 Food policies and programs	3.1 Child feeding practices
1.2 Fortified foods	2.2 Food in schools and beyond	3.2 Food and nutrition education
1.3 Specialized foods	2.3 Food labelling and marketing	3.3 Food and feeding data

**Table 2:** Coverage of policies related to the prevention of childhood overweight and obesity across GCC countries ^1^.

Country/Policy *	UAE	Bahrain	Saudi Arabia	Oman	Qatar	Kuwait
National policies on prevention of overweight and obesity						
Baby-friendly Hospital Initiative						
Regulations on marketing of breastmilk substitutes and complementary foods						
School nutrition environment	Voluntary	Voluntary			Voluntary	Voluntary
Food-based dietary guidelines						
Taxation on sugar-sweetened beverages						
Front-of-pack labelling	Voluntary (Nutri-Mark)		Voluntary (traffic light)			
Menu labelling	Voluntary	Voluntary	Mandatory		Mandatory	
Nutrient profiling system						
Regulations on marketing of food and non-alcoholic beverages to children (broadcast and non-broadcast media)			Voluntary	Voluntary- restrictions during children’s programs on television and radio		Under consideration—pledge only
Restrictions on promotion of unhealthy food in children’s settings	Voluntary	Voluntary	Voluntary—draft under review	Voluntary	Voluntary—draft under review	Voluntary
WHO Acceleration Plan to STOP Obesity—Roadmap						

^1^ Of the policies recommended in the Commitment to the Acceleration Plan to STOP Obesity, WHO NCD BEST Buys, and UNICEF programming guidance. * Policy-status categories are adapted from the UNICEF report [23]. Dark blue shading denotes reported full existence and implementation; blue shading denotes reported partial implementation; light blue and gray denotes that the source report identified no policy as available. These are descriptive classifications from the source report, which did not specify standardized implementation thresholds. Mandatory or voluntary status is indicated separately where documented. Shading should not be interpreted as independent verification of compliance or effectiveness. Abbreviations: NCD, noncommunicable disease; UAE, United Arab Emirates; UNICEF, United Nations Children’s Fund; WHO, World Health Organization.

**Table 3:** Priority recommendations to strengthen childhood overweight and obesity prevention efforts in the GCC region.

Action Area 1: Improving Children’s Foods	Action Area 2: Improving Children’s Food Environments	Action Area 3: Improving Children’s Food Practices
Adopt regional food-based dietary guidelines that address the nutrition rights and needs of children, adolescents and women	Strengthen policies already in use, including taxes on SSB and front-of-pack labeling	Mandate implementation and monitoring frameworks for all obesity-related policies, including school nutrition, physical activity, and breastfeeding initiatives
Set GCC nutrient-profile criteria and reformulation targets for foods and beverages commonly consumed by children, limiting added sugars, sodium, and UPF	Implement comprehensive restrictions on marketing of unhealthy foods and beverages to children, including digital marketing	Develop community engagement programs that support families in preparing healthy foods for infants, children, and adolescents, with practical and culturally appropriate guidance on meal planning, food preparation, and age-appropriate feeding
Set GCC quality standards for commercial complementary, fortified, and specialized foods for children, with product-composition monitoring and limits on added sugars and salt.	Mandate and enforce the regulation of school food standards and procurement rules, including bans on sugary drinks and UPF in school premises.	Mandate health insurance companies to cover breastfeeding counseling, consultations, and devices within postpartum care packages.
		Optimize the design and implementation of information, communication, and counselling programs that support breastfeeding and complementary feeding counselling.

## Data Availability

The data presented in this study are available on request from the corresponding author. The recordings are not publicly available to protect participants’ privacy and confidentiality and minimize the risk of participant identification.

## Supplementary Materials

The following supporting information can be downloaded at: https://www.mdpi.com/article/10.3390/nu18203435/s1, Table S1: Policy-related documents across key domains relevant to childhood overweight and obesity prevention and management in the GCC (*n* = 103); Table S2: Country-level summary of documented measures across five domains relevant to childhood obesity prevention.

## Abbreviations

The following abbreviations are used in this manuscript:

TLA	Three letter acronyms
AI	Artificial intelligence
GCC	Gulf Cooperation Council
GSO	GCC Standardization Organization
Gulf CDC	Gulf Center for Disease Prevention and Control
IYCF	Infant and young child feeding
MENA	Middle East and North Africa
NCDs	Noncommunicable diseases
SDG	Sustainable Development Goal
SSBs	Sugar-sweetened beverages
UAE	United Arab Emirates
UNESCO	United Nations Educational, Scientific and Cultural Organization
UNICEF	United Nations Children’s Fund
UPFs	Ultra-processed foods
WHO	World Health Organization
