# Supplementary material for: Transforming Food Systems for the Prevention of Childhood Overweight and Obesity in the Gulf Region
Source: Nutrients. 2026 Oct 20;18(20):3435. doi: 10.3390/nu18203435 (PMC13708571; doi:10.3390/nu18203435)
Supplement: Supplementary file 1 [file nutrients-18-03435-s001.zip › nutrients-4549657-supplementary.pdf]

Table S1. Policy-related documents across key domains relevant to childhood overweight and obesity prevention and management in the GCC (n=103)

Country/ region	Document Title	Year	Issuing Body	Key Domain(s)
UAE (n=16)	We the UAE 2031	2022	Government of UAE	Youth/Family Engagement; Social Determinants of Health/Urban Planning
	UAE Circular Economy Policy	2021-2031	Ministry of Cabinet Affairs	Social Determinants of Health/Urban Planning
	National Multisectoral Plan to Combat NCDs and Associated Risk Factors	2017-2021	MOHAP	Obesity Prevention
	The National Policy for Promoting Healthy Lifestyles	2022-2026	MOHAP	Diet and Nutrition; Obesity Prevention; Physical Activity
	National Plan to Control Noncommunicable Diseases and Associated Risk Factors	2023-2026	MOHAP	Obesity Prevention
	National Health and Well-being Strategic Framework	2023-2031	National Program for Happiness & Well-being	Obesity Prevention; Social Determinants of Health/Urban Planning
	National Monitoring Framework for the Prevention and Control of NCDs	2024	MOHAP	Obesity Prevention; Monitoring and Evaluation
	National Nutrition Strategy	2023-2031	MOHAP	Diet and Nutrition
	Labeling of Prepackaged Food Stuffs (ICS-2017)	2017	ESMA	Diet and Nutrition; Food Environment
	Nutritional Labelling of Pre-Packed Products as Traffic Light Colors	2018	ESMA	Diet and Nutrition; Food Environment
	Permitted Health and Nutrition Claims Made on Food and their Conditions of Use	2018	ESMA	Diet and Nutrition; Food Environment
	The National Youth Agenda 2031	2016	Ministry of Youth Affairs	Youth/Family Engagement; Social Determinants of Health/Urban Planning
	A Guide to Food Security in the UAE	2023	Ministry of Climate Change and Environment	Diet and Nutrition; Social Determinants of Health/Urban Planning
	National Plan to Combat Childhood Obesity	2017	MOHAP	Obesity Prevention; Youth/Family Engagement
	National Plan to Combat Diabetes, Obesity and Related Risk Factors	2022-2026	MOHAP	Obesity Prevention
	The National Policy for the Promotion of Women's Health in the UAE	2024	MOHAP	Breastfeeding/IYCF; Obesity Prevention; Youth/Family Engagement
Bahrain (n=22)	Economic Vision 2030	2008	Government of Bahrain	Youth/Family Engagement; Social Determinants of Health/Urban Planning
	National Health Strategy	2016-2025	Supreme Council of Health	Obesity Prevention

	Health Promotion Strategy	2023-2026	MOH	Obesity Prevention; School/Education Setting; Youth/Family Engagement
	National Action Plan for Control and Prevention of NCDs and Associated Risk Factors	2019-2025	MOH	Obesity Prevention
	National Strategy for Physical Activity and Healthy Diet	2018	MOH	Diet and Nutrition; Obesity Prevention; Physical Activity
	Breastfeeding Strategies	2018	MOH	Diet and Nutrition; Breastfeeding/IYCF
	Obesity Prevention in Schools	2018	MOE	Obesity Prevention; School/Education Setting
	National Plan to Control Child and Adolescent Obesity	2021	MOH	Obesity Prevention; Youth/Family Engagement
	National Plan for Nutrition for Infants and Children	2022	MOH	Diet and Nutrition; Breastfeeding/IYCF; Youth/Family Engagement
	Obesity Action Acceleration Roadmap	2023	MOH	Obesity Prevention
	Weight Management Clinic Program	2023	MOH	Obesity Prevention; Obesity Management
	Food National Based Dietary Guidelines	2023	MOH	Diet and Nutrition
	Pre-import Licensing for Labeling and Health and Quality Claims - Special Food Guidelines	2022	MOH	Diet and Nutrition; Food Environment
	Regulating the Marketing and Promotion of Breast-Milk Substitutes	2018	MOH	Food Environment; Breastfeeding/IYCF; Youth/Family Engagement
	Regulations of Energy Drinks Within the Executive Regulations of the Public Health Law (Resolution No. 13)	2021	Ministry of Industry and Commerce	Diet and Nutrition; Food Environment; Youth/Family Engagement
	Mandatory Requirements of the National Guideline of Food and Drinks Provided in School Canteens (Law Provision No. 10)	2024	MOH	Diet and Nutrition; Food Environment; School/Education Setting
	Vending Machines Initiative: Making Healthy Choices the Easier Option	2024	MOH	Diet and Nutrition; Food Environment; Obesity Prevention
	Action Plan for the Ministry of Education	2022-2023	MOH	School/Education Setting; Social Determinants of Health/Urban Planning
	Action Plan for the Ministry of Works, Municipalities Affairs, and Urban Planning	2023-2026	MOH	Social Determinants of Health/Urban Planning
	Action Plan for Industry and Commerce	2019-2025	MOH	Diet and Nutrition; Food Environment; Social Determinants of Health/Urban Planning
	Action Plan for the Supreme Council for Environment	2019-2025	MOH	Social Determinants of Health/Urban Planning
	Action Plan for the Ministry of Information for Health Promotion	2024	MOH	Obesity Prevention; Youth/Family Engagement
Saudi Arabia (n=17)	Saudi Vision 2030	2016	Government of Saudi Arabia	Youth/Family Engagement; Social Determinants of Health/Urban Planning

	KSA National Strategy for Prevention of NCDs	2014-2025	MOH	Obesity Prevention
	National Executive Plan for NCDs (Diabetes Control)	2014-2025	MOH	Obesity Prevention
	High-Impact Interventions to Reduce NCD Standardized Mortality Rate	2024-2030	PHA	Obesity Prevention
	Obesity Control Program Strategy	2014-2025	MOH	Obesity Prevention; Obesity Management
	Healthy Food Strategy	2018-2030	SFDA	Diet and Nutrition; Food Environment; Obesity Prevention
	Obesity Prevention and Control Strategy	2020-2023	PHA	Obesity Prevention; Obesity Management
	Saudi Guideline on the Prevention and Management of Overweight and Obesity	2022	MOH	Obesity Prevention; Obesity Management
	National Strategy for Diet and Physical Activity	2014-2025	SFDA	Diet and Nutrition; Obesity Prevention; Physical Activity
	Guidelines of Marketing Requirements of Food Directed to Children	2023	SFDA / GCC Standardization Organization	Diet and Nutrition; Food Environment; Obesity Prevention; Youth/Family Engagement
	Implementing Regulations of the Law on Trading of Breastfeeding Substitutes	2014	MOH	Food Environment; Breastfeeding/IYCF; Youth/Family Engagement
	Health Requirements for Providing School Nutrition Services	2013	MOE	Diet and Nutrition; Food Environment; School/Education Setting
	Preventing Unhealthy Food in School Canteens and Cafeterias	2017	MOE	Diet and Nutrition; Food Environment; Obesity Prevention; School/Education Setting
	Twenty-Four-Hour Movement Practice Guidelines for Saudi Arabia: An Integration of Physical Activity, Sedentary Behavior, and Sleep Duration	2021	PHA	Obesity Prevention; Physical Activity; Sedentary Behavior
	Allowing Physical Exercise in Girls' Schools in the Kingdom	2018	Quality of Life Program	Obesity Prevention; Physical Activity; School/Education Setting; Youth/Family Engagement
	My Sport is My Future Program	2018	MOE	Obesity Prevention; Physical Activity; School/Education Setting; Youth/Family Engagement
	General Strategy for Sports	2021	Saudi Sports for All Federation	Obesity Prevention; Physical Activity
Oman (n=8)	Oman Vision 2040	2019	Government of Oman	Youth/Family Engagement; Social Determinants of Health/Urban Planning
	National Multisectoral Action Plan for the Prevention and Control of NCDs	2016	MOH	Obesity Prevention
	National Policy for the Prevention and Control of Non-Communicable Diseases	2016-2025	MOH	Obesity Prevention
	National Monitoring Framework for Prevention and Control of NCDs	2020-2030	MOH	Obesity Prevention; Monitoring and Evaluation

	National Nutrition Strategy and Framework for Action	2020-2030	MOH	Diet and Nutrition; Breastfeeding/IYCF
	Obesity in Oman Briefing Book	2021	MOH	Obesity Prevention; Obesity Management
	Policy Implications for the Prevention of Female Obesity in Oman	2023	MOH	Obesity Prevention; Youth/Family Engagement
	Guide to the Healthy Villages Program	2018	MOH	Youth/Family Engagement; Social Determinants of Health/Urban Planning
Qatar (n=20)	Qatar National Vision 2030	2008	Government of Qatar	Youth/Family Engagement; Social Determinants of Health/Urban Planning
	Qatar Public Health Strategy	2017-2022	MOPH	Obesity Prevention
	Healthy Public Policy	2018	MOPH / Health in All Policies	Obesity Prevention; Social Determinants of Health/Urban Planning
	Healthy Cities Program Qatar	2018	MOPH / WHO / Gulf Health Council	Obesity Prevention; Social Determinants of Health/Urban Planning
	National Health Strategy	2024-2030	MOPH	Obesity Prevention
	The National Obesity, Diabetes, and Modifiable Risk Factors for Atherosclerotic Cardiovascular Diseases (ASCVD) Action Plan	2024-2030	MOPH	Diet and Nutrition; Obesity Prevention
	National Diabetes Strategy	2016-2022	MOPH	Obesity Prevention
	Policy Framework: Case Studies of Successful Childhood Obesity Policies Around the World	2019	MOPH	Obesity Prevention; Youth/Family Engagement
	Childhood Obesity in Qatar: National Policy Proposal through Multisectoral Approaches	2023	World Innovation Summit for Health and MOPH	Obesity Prevention; Youth/Family Engagement; Social Determinants of Health/Urban Planning
	Qatar National Nutrition and Physical Activity Action Plan	2017-2022	MOPH	Diet and Nutrition; Obesity Prevention; Physical Activity
	The Qatar Dietary Guidelines	2022	MOPH	Diet and Nutrition
	Food and Beverage Labelling at Restaurants and Coffee Shops	2018	Ministry of Commerce and Industry / MOPH	Diet and Nutrition; Food Environment
	Qatar National Physical Activity Guidelines - 2nd Edition	2021	Aspetar Orthopedic and Sports Medicine Hospital	Obesity Prevention; Physical Activity
	Open Space, Recreation and Sport Facilities Development Guidelines	2017	MOPH / Ministry of Municipality	Obesity Prevention; Physical Activity; Social Determinants of Health/Urban Planning
	Qatar Olympic Committee Strategy	2023-2030	The Qatar Olympic Committee	Obesity Prevention; Physical Activity
	Qatar Sports Strategy	2023-2030	The Qatar Olympic Committee	Obesity Prevention; Physical Activity

	Qatar National Housing Strategy	2023	Ministry of Municipality and Environment	Social Determinants of Health/Urban Planning
	Qatar Urban Design Compendium Project	2019	Ministry of Municipality and Environment	Social Determinants of Health/Urban Planning
	Qatar Planning Legislation	2024	Ministry of Municipality and Environment / MOPH	Social Determinants of Health/Urban Planning
	Education Strategy	2024-2030	Ministry of Education and Higher Education	School/Education Setting; Youth/Family Engagement; Social Determinants of Health/Urban Planning
Kuwait (n=16)	New Kuwait 2035	2017	Government of Kuwait	Youth/Family Engagement; Social Determinants of Health/Urban Planning
	White Paper for the Health Pillar - The National Health Policy Framework for Kuwait	2019	Supreme Council for Planning and Development	Obesity Prevention
	National Strategy for the Prevention and Control of Chronic NCDs	2020-2030	MOH	Obesity Prevention
	National Policy for the Prevention and Control of NCDs	2025	MOH	Obesity Prevention
	Kuwait Food-Based Dietary Guidelines: National Guidelines for a Healthy Diet	2023	PAFN	Diet and Nutrition
	Obesity Clinical Guidelines in Chronic Disease in Primary Healthcare	2022	MOH	Obesity Prevention; Obesity Management
	Clinical Nutrition Guidelines for Pediatric Obesity	2023	MOH	Diet and Nutrition; Obesity Prevention; Obesity Management; Youth/Family Engagement
	Ministerial Resolution No. (5) of 2021 (School Canteens Regulation)	2021	PAFN	Diet and Nutrition; Food Environment; School/Education Setting
	Healthy Canteens Project	2024	PAFN	Diet and Nutrition; Food Environment; Obesity Prevention; School/Education Setting
	Added Sugar Reduction Initiative	2024	PAFN	Diet and Nutrition; Food Environment
	Food Packaging Facade Labelling Initiative Guide in the Form of Traffic Lights, Front of Pack Nutrition Labelling Initiative	2024	PAFN	Diet and Nutrition; Food Environment
	Kuwait's National Strategy and Action Plan on Physical Activity	2021-2026	MOH	Obesity Prevention; Physical Activity
	Guidelines on Physical Activity and Sedentary Behavior	2024	MOH	Obesity Prevention; Physical Activity; Sedentary Behavior
	Physically Active Schools Initiative	2020	MOH	Obesity Prevention; Physical Activity; School/Education Setting

	Urban Design Manual	2019	Public Authority for Housing Welfare	Social Determinants of Health/Urban Planning
	Implementation of the Healthy Cities Program	2022	Health Cities Office / MOH	Obesity Prevention; Social Determinants of Health/Urban Planning
GCC/ Regional (n=4)	UNICEF MENA Nutrition Strategic Direction	2024	UNICEF	Diet and Nutrition; Obesity Prevention
	A Report on Transforming Food Systems for the Prevention of Childhood Overweight in Gulf Countries	2026	UNICEF/WHO/Gulf-CDC	Diet and Nutrition; Obesity Prevention; Social Determinants of Health/Urban Planning
	WHO EMRO Nutrition Strategy	2020–2030	WHO EMRO	Diet and Nutrition; Obesity Prevention
	WHO EMRO Policy Priorities for Obesity & Diabetes	2017	WHO EMRO	Diet and Nutrition; Obesity Prevention; Physical Activity; Sedentary Behavior

ASCVD, Atherosclerotic Cardiovascular Diseases; EMRO, Eastern Mediterranean Regional Office; ESMA, Emirates Authority for Standardization and Metrology; GCC, Gulf Cooperation Council; Gulf CDC, Gulf Center for Disease Prevention and Control; ICS, International Classification for Standards; IYCF, Infant And Young Child Feeding; KSA, Kingdom of Saudi Arabia; MENA, Middle East and North Africa; MOE, Ministry of Education; MOH, Ministry of Health; MOHAP, Ministry of Health and Prevention; MOPH, Ministry of Public Health; NCD(s), Non-Communicable Disease(s); PAFN, Public Authority for Food and Nutrition; PHA, Public Health Authority; SFDA, Saudi Food and Drug Authority; UAE, United Arab Emirates; UNICEF, United Nations Children's Fund; WHO, World Health Organization.

Table S2. Country-level summary of documented measures across five domains relevant to childhood obesity prevention.

Domain	Findings	Countries	Notes
**School nutrition**	School canteen standards or guidelines documented in 6/6 countries (100%).	Standards/guidelines: all six.	Countries also have voluntary components. These findings do not mean that the entire school nutrition programme is mandatory or fully implemented.
**Food marketing to children**	UNICEF's policy table identifies voluntary broadcast/non-broadcast marketing measures in 2/6 countries (33.3%), with a proposal or pledge under consideration in 1/6 (16.7%).	Voluntary: Saudi Arabia and Oman.<br><br>Under consideration: Kuwait.	These are classifications based on the UNICEF report, not a complete assessment of subsequent national adoption or enforcement.
**Front-of-pack labelling**	Voluntary schemes identified in 3/6 countries (50%).	UAE, Saudi Arabia, and Oman	There is a Kuwaiti initiative guide.
**Infant and young child feeding**	The Baby-friendly Hospital Initiative is documented in 6/6 countries (100%).	All six.	Presence of this initiative does not establish mandatory status, coverage of all facilities, or evaluated feeding outcomes.
**Physical activity**	At least one relevant policy, strategy, guideline, plan or programme documented in 6/6 countries (100%).	All six.	

Counts use six GCC countries as the denominator and describe measures documented in the source reports. Mandatory and voluntary components may coexist within the same country and domain. Policy presence does not establish implementation, enforcement or effectiveness.
